# *Helicobacter pylori*-Derived Outer Membrane Vesicles (OMVs): Role in Bacterial Pathogenesis?

**DOI:** 10.3390/microorganisms8091328

**Published:** 2020-08-31

**Authors:** Miroslaw Jarzab, Gernot Posselt, Nicole Meisner-Kober, Silja Wessler

**Affiliations:** 1 Division of Microbiology, Department of Biosciences, Paris-Lodron University of Salzburg, 5020 Salzburg, Austria; miroslaw.jarzab@sbg.ac.at (M.J.); gernot.posselt@sbg.ac.at (G.P.); 2 Division of Chemical Biology and Biological Therapeutics, Department of Biosciences, Paris-Lodron University of Salzburg, 5020 Salzburg, Austria; nicole.meisner-kober@sbg.ac.at; 3 Cancer Cluster Salzburg, Allergy-Cancer-BioNano Research Centre and, Paris-Lodron University of Salzburg, 5020 Salzburg, Austria

**Keywords:** CagA, *Helicobacter pylori*, HtrA, outer membrane vesicles, OMV, urease, VacA

## Abstract

Persistent infections with the human pathogen *Helicobacter pylori* (*H. pylori*) have been closely associated with the induction and progression of a wide range of gastric disorders, including acute and chronic gastritis, ulceration in the stomach and duodenum, mucosa-associated lymphoid tissue (MALT) lymphoma, and gastric adenocarcinoma. The pathogenesis of *H. pylori* is determined by a complicated network of manifold mechanisms of pathogen–host interactions, which involves a coordinated interplay of *H. pylori* pathogenicity and virulence factors with host cells. While these molecular and cellular mechanisms have been intensively investigated to date, the knowledge about outer membrane vesicles (OMVs) derived from *H. pylori* and their implication in bacterial pathogenesis is not well developed. In this review, we summarize the current knowledge on *H. pylori*-derived OMVs.

## 1. Background

*Helicobacter pylori* (*H. pylori*) is a Gram-negative human pathogen that specifically colonizes the epithelial lining in the human stomach. Persistent infections with *H. pylori* are invariably associated with a mild chronic inflammation of the gastric mucosa, which often remains asymptomatic [1]. However, a significant proportion of infected individuals develop clinically relevant symptoms of gastritis, gastric and duodenal ulcers, mucosa-associated lymphoid tissue (MALT), or gastric cancer [2,3]. Consequently, *H. pylori* was classified as the first bacterial group-I carcinogen by the World Health Organization in 1994 [4]. It is estimated that in connection with environmental influences, genetic predispositions of the host or microbiota, *H. pylori* represents the primary cause of more than 90% of gastric cancer cases [5,6]. *H. pylori*-dependent gastric adenocarcinomas develop through a series of sequential transformations of the gastric mucosa, which is known as the ’Correa cascade´ [7]. This process is initiated by chronic gastritis, leading to atrophic gastritis, intestinal metaplasia, dysplasia, and finally to carcinoma [8]. Eradication of *H. pylori* with antibiotics has been demonstrated to be an effective strategy for preventing cancer development and progression [9,10].

*H. pylori* developed a number of survival stratagems that allow bacterial persistence and immune evasion despite severe pathogenesis. In this context, *H. pylori* expresses a wide range of niche and virulence factors (reviewed in [11,12,13]), which are implicated in a complex molecular network that controls the induction and progression of *H. pylori*-dependent disorders [14]. Outer membrane vesicles (OMVs) provide an emerging mechanism to interfere with host cell physiology. Membrane vesicles are ubiquitously produced among organisms from all domains of life, including prokaryotes, eukaryotes and archaea [15]. Mammalian cells secrete extracellular vesicles (EVs), derived from both the plasma membrane (microvesicles or ectosomes) and from intracellular endosome-derived multivesicular bodies (exosomes), exhibiting manifold functions in health and disease [16]. Membrane vesicles from Gram-negative bacteria are termed OMVs to emphasize their origin from the outer membrane [17]. Functionally, OMVs are implicated in intermicrobial communication through exchanges of genetic material, chemical compounds and signaling molecules [18]. Nevertheless, several publications also suggest that OMVs play a direct role in bacterial pathogenesis [19,20,21,22,23]. In this review, we will summarize the current state of research on *H. pylori*-derived OMVs.

## 2. Physicochemical Characteristics of *H. pylori*-Derived OMVs 

The physical interaction between *H. pylori* and gastric epithelial cells has been intensively investigated over the last 30 years, and has revealed a complex network of manifold cellular and molecular mechanisms, which are coordinated in time and place [12,24,25]. *H. pylori*-derived OMVs add a new aspect to the pathogenesis of *H. pylori*. OMVs of *H. pylori* were analyzed for the first time in 1997 by dual silver staining [26]. Since then, *H. pylori* OMVs have been studied both in vivo and in vitro in gastric biopsies [19,27,28], in gastric juice samples of infected individuals [29], and in culture media of *H. pylori* [19,27,28,30,31,32], especially during the late stationary growth phase and during biofilm formation [30,33,34,35,36]. A standard methodology in the EV field is Nanoparticle Tracking Analysis (NTA), which uses a light diffraction-based detection of particles diffusing through an observation volume, and sizing based on translational diffusion analysis of the Brownian motion, allowing the detection of particles >50 nm. An unbiased size determination is currently only possible by electron microscopy (EM) or atomic force microscopy (AFM) [37]. Generally, bacterial OMVs are thought to be relatively stable structures. Long-term storage has not been studied extensively, but one study reported that OMVs from *H. pylori* J99 or NCTC 11637 (ATCC 43504) stored at 4 °C overnight or at −20 °C did not differ in their macroscopic structure, resembling a deformed football [38]. Usually, OMVs from different *H. pylori* strains range between 20 and 300 nm in size, but some studies reported diameters of 400 nm to 500 nm (Table 1). Size analyses also suggest that OMV diameters change with bacterial growth phase. Vesicles isolated from bacteria grown to early logarithmic phases were larger compared to vesicles derived from bacteria in the stationary phase [39]. OMVs in this study were examined by NTA, and therefore it is possible that vesicles even smaller than 50 nm are produced but not detected. Whether the observed OMV size differences are specific for different *H. pylori* strains, and/or are the result of differing culture and assay conditions, isolation methods and vesicle analysis methods, needs to be investigated in the future. A systematic and technically unbiased evaluation of *H. pylori* vesicle sizes might, however, be particularly relevant to advancing our understanding of OMV function, since it has been implied that OMV size influences host cell entry and protein content [40]. In addition to the size, the number of OMVs released by *H. pylori* also depends on bacterial growth and environmental conditions. The release of *H. pylori* OMVs is inversely related to the cell growth, shifting from low quantities of OMVs released during the logarithmic growth phase to high levels produced during the stationary phase. This process is also correlated with the shape transitions of the bacterium from spiral to curved, doughnut-shaped and finally coccoid morphologies [41]. The number of OMVs secreted also changes with culturing conditions or environmental factors, which the bacteria encounter during the infection process, as well as bacterial mutations. A direct comparison of *H. pylori* 251 strain and an isogenic mutant exhibiting a deletion in the *“colicin”-tolerant* (*tol*) B (HP1126) locus revealed a more than 600-fold increase in OMV concentrations, while the protein composition characterized by LC/MS-proteomics was largely overlapping [42]. In *H. pylori*, the HP1126 locus is not well understood, but in *Escherichia coli* (*E. coli*), TolB is a periplasmic protein interacting with outer membrane proteins (OMPs) and peptidoglycan-associated lipoprotein (Pal), which was originally identified to play a role in the import of a number of colicins [43]. Disruptions in *tol-pal* clusters can result in defects in the growth, motility and virulence of *E. coli*, as well as of other Gram-negative bacteria [44]. Hence future studies are necessary to investigate the impact of HP1226 on OMV biogenesis in *H. pylori* hyper-vesiculating strains.

## 3. *H. pylori* OMVs Promote Biofilm Formation

Biofilms are surface-associated communities of bacteria that are embedded in a hydrated matrix of extracellular polymeric substances. Biofilm formation helps bacterial cells to avoid antimicrobials and protects them from being killed by the host’s immune system, thus enabling chronic infections. The bacteria in biofilms are phenotypically distinct from their planktonic counterparts. Not much is known about *H. pylori’s* ability to develop biofilms, its structure and composition, or the genes associated with this mode of growth [45]. However, several studies have demonstrated the ability of *H. pylori* to produce a biofilm in vitro [30,33,34,35,46]. In addition, there are also some reports on biofilm formation during the colonization of the human gastric mucosa with *H. pylori*. In SEM analyses of biopsy specimens, the presence of dense layers resembles biofilm-associated bacteria at the mucosal surface of *H. pylori*-positive patients [47,48].

In vitro studies of *H. pylori* biofilm formation indicate an involvement of OMVs in this process; however, it is still unclear which molecular and cellular mechanisms are involved. One study reported that the biofilm formation ability of *H. pylori* was dependent on OMVs mediating direct cell-to-cell contacts between the bacteria. The addition of OMVs, from a clinical isolate (TK1402) with strong biofilm-forming activity, to *H. pylori* cultures significantly enhanced biofilm formation ability [35]. An unidentified 22 kD protein [33] and an omp20 (HP0912, AlpB) protein in OMVs were described as important factors in biofilm formation [34]. Another study has shown that *H. pylori* ATCC 43629/NCTC 11639 OMVs isolated from a 48 h biofilm contained fourfold extracellular DNA (eDNA), compared to OMVs isolated from planktonic cultures of the same strain, indicating that OMVs may prevent eDNA degradation, which could additionally support or modulate biofilm formation or play a role in the intercellular exchange of genetic material. *H. pylori* (ATCC 43629/NCTC 11639) OMVs isolated directly from biofilms (bOMVs) showed a broader size range between 10 nm and 2.5 µm, with a major peak at 681.1 nm (59%) and two minor populations at 1760 nm (29.9%) and 2557 nm (11%) [30]. The enzyme carbonic anhydrase was also identified, and it was suggested that carbonic anhydrase and dsDNA released into the medium could stabilize the biofilm formation [36]. The change in the content of isolated OMVs during the development of biofilms was also observed in other *H. pylori* strains [33]. Despite individual reports investigating *H. pylori* biofilms, a significant role for biofilms in *H. pylori* survival and pathogenesis has yet to be determined. 

## 4. The Content of *H. pylori* OMVs

In several reports, the content of isolated OMVs was analyzed, and large sets of lipids, nucleic acids, peptidoglycan and proteins were identified (Table 2). Dual silver staining revealed an increased ratio of carbohydrate to protein in OMVs compared to whole bacterial cells [26,27]. Protein composition as well as the carbohydrate to protein ratio of OMVs differ between bacterial strains [26]. Differences in the size and density of OMVs are also reflected in differences in vesicle composition. The high density vesicles contain higher protein to lipid ratios. Furthermore, differences in environmental conditions affect the content of vesicles [41] and the bacterial growth stage influences the abundance of key virulence determinants in OMVs [39]. 

### 4.1. Lipids, Peptidoglycan, and Nucleic Acids in H. pylori OMVs 

Analysis of the membrane lipids provides information about OMV biogenesis since the inner membrane (IM) differs from outer membrane (OM) and OMVs in phospholipid content. Consistent with the biogenesis of OMVs, the phospholipid composition of OMVs closely resembles that of the OM [41]. IM contains lower amounts of phosphatidylglycerol and lyso-phosphatidyletanolamine, compared to OM and OMVs membrane fractions. In turn, phosphatidylcholine was solely detected in OM and OMVs. Phosphatidylethanolamine and cardiolipin were detected as major phospholipids in whole cell extracts (WCE), OM and IM, and purified OMVs. Significant amounts of cholesterol were also detected in WCE as well as in OMVs. Using ^13^C, ^1^H NMR correlation spectra analysis, it was estimated that the cholesterol content is about 10% of total phospholipids present in both WCE and OMVs [41], which is generally lower than the cholesterol fraction reported for mammalian EVs [49]. *H. pylori* lipopolysaccharide (LPS) is enriched in OMVs compared to OM [26,50], and is influenced by environmental conditions. LPS levels in *H. pylori* and OMVs are reduced in iron-limited conditions, probably due to the decreases in energy supply indicated by lower ATP levels [50].

In addition to dsDNA, several studies have shown the presence of small RNAs in *H. pylori* OMVs, and suggested a functional role upon their transfer into host cells [51,52]. More than a half million unique small non-coding (snc) RNA sequences were identified by RNA seq of OMVs purified from the supernatants of *H. pylori* strain J99, and RNAse protection assays for selected sncRNAs suggested an encapsulation within the OMV lumen [53]—or at least a lower susceptibility to digestion due to OMV binding and protection by a protein corona on the OMV surface, as has been suggested for small RNAs in mammalian EVs [54,55]. The uptake of sncRNAs into AGS cells was suggested by quantitative RT- PCR and microscopy using double labeling with a lipid dye (DiI) and an unspecific RNA intercalating dye. Of note, the lipid labeling of mammalian EVs can easily give rise to a number of artefacts, including micelle formation, unspecific labeling of other particles within the heterogeneous samples and the secondary redistribution of the dye, and it is conceivable that similar issues also apply for OMVs. Nevertheless, this study reports a functional effect of the *H. pylori* OMV on LPS-stimulated interleukin-8 (IL-8) secretion by gastric epithelial AGS cells, and has convincingly attributed it to two candidate small RNAs identified in the OMV transcriptome, and the authors hypothesized that these small RNAs might target the human IL-8 mRNA directly [53]. While more studies are required, these data suggest that small RNAs within *H. pylori* OMVs might play a fundamental role in directly tuning the host immune response.

### 4.2. The Protein Content of H. pylori OMVs 

**Figure 1:**
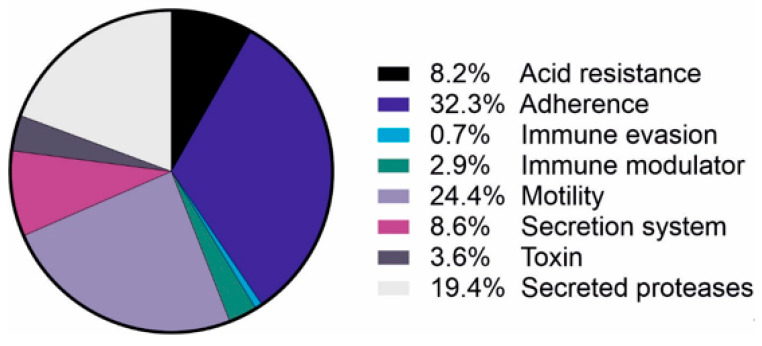
Virulence factors detected in *H. pylori*-derived OMVs. Identified virulence factors of *H. pylori* by mass spectroscopy [38,39,40,41,42,45] and their role in bacterial pathogenesis.

*H. pylori* OMVs exhibit a large heterogeneity in their protein composition. Interestingly, in contrast to the lipid composition, the proteome of OMVs significantly differs from whole cells and periplasm, suggesting a regulated sorting mechanism as is well known for mammalian EVs [38,41]. OMVs are predominantly enriched in uncharacterized proteins, flagellar basal body proteins [39] or outer membrane proteins (OMPs) (Table 2) [41]. More than 500 different *H. pylori* proteins were identified using mass spectroscopy analyses. However, most available reports show conflicting data, most likely due to the unstandardized sample preparation and analysis protocols. Nevertheless, the heterogeneity of OMV proteins is influenced by strain type, different culture conditions and bacterial growth phase [23]. Consistently identified targets include the proteins involved in cellular processing and signaling (especially cell motility and cell wall, membrane or envelope biogenesis), metabolism (energy production and conversion), and several well-known *H. pylori* virulence factors. Proteins identified in OMVs and considered as virulence factors (Table 2) belong to the group of proteins implicated in adherence, motility, acid resistance, immune evasion and modulation, and protein secretion of proteins (Figure 1). 

#### Uptake of *H. pylori* OMVs: Delivery of Virulence Factors into Host Cells? 

**Figure 2:**
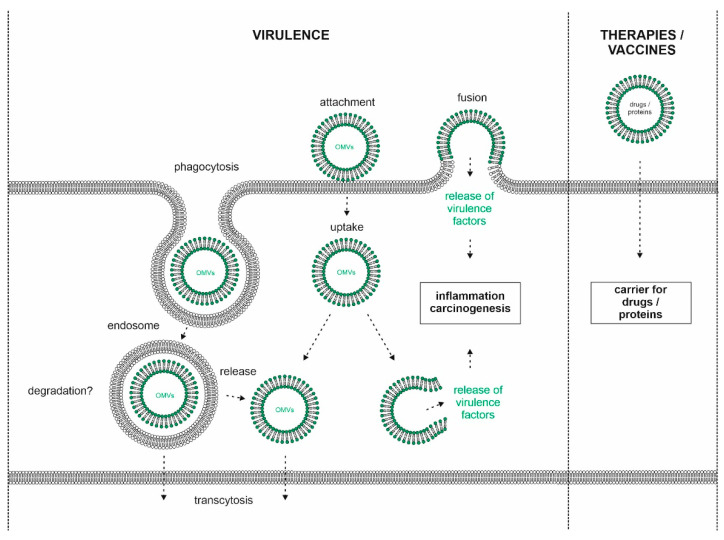
Model of OMV interaction with host cells. *H. pylori* releases OMVs, which attach to the cell surface of gastric epithelial cells. Clathrin-dependent and -independent uptake of OMVs could occur via phagocytosis leading to the formation of endosomes and/or fusion of the bacterial OMV membrane with the host´s membrane. The fate of internalized OMVs is unclear, but the release of OMVs at the basolateral membrane (transcytosis) or into the cytoplasm was hypothesized. OMV-delivered virulence factors might be released into the cytosol where they can interfere with host cell signaling leading to inflammation and carcinogenesis (left panel). Technical applications of *H. pylori* OMVs as nanocarriers for drugs or proteins (right panel).

It has been reported that orally administered OMVs remain inside the stomach of mice for at least 24 h and can enter gastric epithelial cells [29]. Additionally, OMVs attach to, and internalize into, primary human antrum cells [31]. Clathrin-dependent and clathrin-independent mechanisms are described for OMV internalization, resulting in a highly efficient and rapid uptake in gastric epithelial AGS cells [56,57] (Figure 2). It has been suggested that caveolin mediates the entry of smaller (20–100 nm) OMVs into AGS cells. Larger OMVs might enter AGS cells via macropinocytosis, caveolin and clathrin-dependent endocytosis without preference [40]. By EM imaging, OMVs were found within the endosomes of human gastric mucosa cells [19,32]. Internalized OMVs colocalize with markers for early endosomes [57,58] and lysosomes, suggesting transport through the endocytic pathway [57] (Figure 2). This is particularly intriguing since it converges with the uptake and trafficking routes of mammalian exosomes within their recipient cells [59]. Once internalized, OMVs are relatively stable at least for 12 h within AGS [29,60], or 72 h within MKN28 cells [32]. Two independent studies reported that the uptake of *H. pylori* OMVs into AGS cells was significantly decreased upon the cell membrane depletion of cholesterol and the disruption of cholesterol-rich lipid rafts [20,57]. OMVs from *H. pylori* strain 60190 did not affect AGS cells’ viability but did influence proliferation; it was indicated that at low doses, OMVs slightly increased [22], and at higher doses, OMVs reduced proliferation significantly [22,60].

OMV proteins involved in *H. pylori* motility comprise a relatively large group (Table 2, Figure 1) and include proteins involved in flagella assembly [61]. *H. pylori* P12 and 18943 OMVs contained catalase (KatA), which is implicated in the protection of bacteria from extracellular ROS in respiratory burst reactions [62]. Among virulence factors, several known and putative adhesins were detected in OMVs. This group of OMV proteins include HopZ and HorB proteins, the blood group antigen binding adhesin (BabA, BabB), sialic acid-binding adhesion (SabA, SabB), *H. pylori* adhesin A (HpaA), and adherence-associated lipoproteins (AlpA, AlpB) (Table 2). The finding of adhesins on OMV surfaces is important since they could mediate the binding and uptake of OMVs into gastric epithelial cells [41].

Urease is important for buffering the acidic environment for the acid-sensitive pathogen, and thus is crucial for successful colonization of the human stomach [63]. Weak signals for urease were detected for OMVs of *H. pylori* 189 and 249 in Western blot analyses [20]. Further, through mass spectroscopy analysis, the urease subunit β was identified in *H. pylori* 7.13 OMVs [45], and both subunits of the active enzyme complex (α and β) were found in *H. pylori* CCUG 17874 [41], NCTC 11637 and J99 [38], 251 OMVs [40], and 26695, independently of growth phase [39]. Urease accessory proteins (UreH, UreG, UreF) were also identified by mass spectroscopy in *H. pylori* OMVs (Table 2). Additional well-known virulence factors of *H. pylori* (e.g., vacuolating cytotoxin A (VacA), cytotoxin-associated gene A (CagA), high temperature requirement A (HtrA), etc.) have been repeatedly identified in OMVs, suggesting that OMVs are involved in the delivery of virulence factors when taken up by host cells.

VacA was identified in OMVs by mass spectroscopy [39,40,41,42,45] and Western blot analysis [27,29,32,38,41,64,65] from many different *H. pylori* strains [19,27,29,32,38,39,40,41,42,45,65,66,67]. VacA is pleiotropic pathogenic factor of *H. pylori*, which is secreted into the bacterial environment. A prominent VacA-mediated phenotype is the excessive formation of massive cell vacuoles harboring both late endosomal and lysosomal markers [68]. Moreover, VacA induces autophagosome formation [69], can induce apoptosis via mitochondrial damage [70], and might also weaken the epithelial barrier by impeding tight junction integrity [71]. VacA was found on the surface of OMVs [19,27,41]. Ultrastructural immunolocalization of VacA in *H. pylori* 60190 OMVs isolated from bacteria culture and in biopsies from infected patients, confirming that this protein is membrane-associated during the growth of *H. pylori* in vitro and in vivo [19,27]. It was further suggested that the uptake of *H. pylori* 60190 OMVs by AGS cells was enhanced by VacA and reduced by LPS [56]. OMVs isolated from the *H. pylori* 60190 strain and its ∆VacA isogenic strain do not differ in morphology and quantity [65]. *H. pylori* 60190 OMVs have been described to induce the vacuolization of HEp-2 cells [27,72] and AGS cells [22], suggesting that VacA is functionally active on OMVs. Even though the presence of OMVs in host cells was not further analyzed in these studies, OMVs were extensively washed to exclude the presence of free soluble VacA, indicating that OMVs might transfer functionally active VacA into the recipient cells. However, the quantification of extracellular VacA revealed that *H. pylori* OMV VacA accounted for about 25% of total VacA, while the remaining was free-soluble VacA. The ratio was similar for both 60190 and CCUG 17874 strains used [73]. Ultrastructural immunocytochemistry confirmed the release of VacA by *H. pylori* both as free-soluble and as OMV-associated toxin; both forms of VacA are taken up by MKN28 cells and are localized inside vacuoles [19,32,73]. On the contrary, when strains 60190 and SS1 were compared, the amount of OMV VacA differed substantially. A significant difference was also seen in the OMV-mediated vacuolization of RK13 rabbit kidney cells, where OMVs from 60190 induced vacuolization in about 49%, and SS1 OMVs induced it in only 13% of cells [65]. *H. pylori* 60190 and CCUG 17874 OMV-associated VacA poorly vacuolate in MKN28 and HeLa cells [73]. Along with the observation that OMV internalization requires cholesterol [20,57], it is interesting that the disruption of lipid rafts by methyl-β-cyclodextrin (MβCD) decreased vacuolization in cells treated with VacA-containing OMVs, pointing to the essentiality of cholesterol in VacA function [56]. It has been discussed that environmental factors can influence VacA levels in OMVs. Iron limitation affects *H. pylori* 60190 growth, and as OMV numbers and OMV protein content increase, VacA decreases to levels similar to those detected for an isogenic VacA deletion mutant [72]. A significant increase in the number of cells with micronuclei was observed in AGS cells upon incubation with OMV from *H. pylori* strain 60190. This process was accompanied by the redistribution of lysosomal iron and a large decrease in cellular glutathione (GSH), both dependent on VacA [60]. The question whether VacA-containing OMVs impact cell survival is not fully answered. Both OMVs from VacA wildtype and a truncated, non-secretable VacA *H. pylori* strain induced a decrease in AGS viability through apoptosis. These OMVs induced caspase-8, -3 and -9 within the host cells, but apoptosis was mitochondria-independent as cytochrome c release was not detected [66]. This is in contrast with reports of soluble VacA, which leads to cytochrome c release [74]. 

**Table 2:** Proteins detected in *H. pylori* OMVs.

Virulence Factors—Acid Resistance					
Gene/Chromosome (NC_000915)	Function/Description		Virulence Factors	Confirmed by Mass Spectroscopy (Reference)	Confirmed by Western Blotting (Reference)
ureH/HP0067	urease accessory proteins, form a complex that acts as a GTP-hydrolysis-dependent molecular chaperone, activating the urease		Urease	[41]	
ureG/HP0068				[39]	
ureF/HP0069				[39,41]	
ureB/HP0072	urease subunit beta	neutralizes the gastric acidity, NH_3_ damages the gastric epithelium		[38,39,40,41,42,45]	[20]
ureA/HP0073	urease subunit alpha			[38,39,40,41,42]	[20]
**Virulence factors—Adherence**					
hopZ/HP0009	Outer membrane protein		HopZ	[38,40,42,45]	
horB/HP0127	Outer membrane protein 4		HorB	[38,39,40,41,45]	
babA/hopS/HP0317/HP1243	Outer membrane protein 9/28, binds with the epithelial cell receptor Leb, mediates bacterial attachment and colonization		Blood group antigen binding adhesins	[39,40,41,42]	[41]
babB/hopT/HP0896	Outer membrane protein 19, babA paralog			[38,39,40,41,42]	
sabB/hopO/HP0722	sabA homologue		Sialic acid binding adhesins	[40,41,42]	
sabA/hopP/HP0725	binds with sialyl-Lex antigen, mediates bacterial attachment and colonization			[38,40,41,42]	[41]
hpaA/HP0797	neuraminyllactose-binding hemagglutinin		*H. pylori* adhesin A	[38,39,40,41,42]	[38]
alpA/hopC/HP0912	Outer membrane protein 20		adherence-associated lipoprotein	[38,39,40,41,42]	
alpB/hopB/HP0913	Outer membrane protein 21			[39,40,41,42]	[34,41]
**Virulence factors—Immune evasion**					
futA/HP0379	alpha-(1,3)-fucosyltransferases, LPS oligosaccharide biosynthesis		Lipopoly-saccharide Lewis antigens	[39]	
futB/HP0651				[41]	
**Virulence factors—Immune modulator**					
napA/HP0243	DNA protection during starvation, activates neutrophils, mast cells and monocytes		Neutrophil-activating protein (HP-NAP)	[39,40,42]	[38]
oipA/hopH/HP0638	Outer membrane protein 13, bacterial adherence to the gastric epithelium, damages mucosal layer, induces apoptosis and IL-8 expression		Outer inflammatory protein	[38,39,41,42]	[38,41]
**Virulence factors—Motility**					
flaB/HP0115	minor flagellin subunit, polymerizes to form the filaments of bacterial flagella	helps bacteria to minimize contact to acidic environment	Flagella	[39,41,42]	
flgI/HP0246	P-ring protein, assembles around the rod			[39,41,42]	
flgL/HP0295	Hook-associated protein 3			[39,41,42]	
flgH/HP0325	L-ring protein, assembles around the rod			[39,45]	
flaA/HP0601	predominant flagellin subunit, polymerizes to form the filaments of bacterial flagella			[38,39,40,41]	
flaG/HP0751	polar flagellin, rotor/switch protein			[39,42]	
fliD/HP0752	filament-capping protein, flagellin folding chaperone, required for the morphogenesis and for the elongation of the flagellar filament			[39,41,42]	
flgE_1/HP0870	hook protein, links the flagellar filament to the drive apparatus in the basal body			[39,40,41]	
flgD/HP0907	basal body rod modification protein, required for flagellar hook formation, scaffolding protein			[39,40,41,42]	
flgE_2/HP0908	flagellar basal body protein			[39,41]	
flgG_1/HP1092	flagellar basal body protein			[39,42]	
flgK/HP1119	first hook-filament junction protein			[39,41,42]	
flgA/HP1477	involved in the assembly process of the P-ring formation			[40,41]	
fliE/HP1557	MS-ring rod junction protein,			[39,40,42]	
flgC/HP1558	rod protein			[39]	
flgB/HP1559	rod protein of flagellar basal body			[39,42]	
flgG_2/HP1585	distal rod protein			[39,42]	
**Virulence factors—Secretion system (Proteins Required for Cag T4SS Activity)**					
cag1/HP0520	membrane protein in T4SS, associated with IL-8 expression induction and CagA delivery to host cells		Cag PAI type IV secretion system	[41]	
cag3/HP0522	defined localization in T4SS apparatus			[39]	
cagX/HP0528	defined localization in T4SS apparatus			[39]	[41]
cagT/HP532	defined localization in T4SS apparatus, core complex protein in T4SS, helps in the translocation of CagA			[39,41]	[41]
cagM/HP0537	defined localization in T4SS apparatus			[39,41]	[41]
cagN/HP0538	localization in T4SS apparatus is not yet defined			[39]	[41]
cagF/HP0543	localization in T4SS apparatus is not yet defined, CagA chaperone			[41]	
cagD/HP0545	localization in T4SS apparatus is not yet defined			[39,42]	
cagA/HP0547	Scaffold/hub protein, oncoprotein, becomes phosphorylated in the host cells, causes cellular proliferation and elongation, induces IL-8 expression		Secreted T4SS effector cytotoxin-associated gene A	[38,39,41,42,45]	[29,38,41]
**Virulence factors—Toxin**					
vacA/HP0887	induces vacuolization of epithelial cells and endoplasmic reticulum stress, causes cell vacuolization, necrosis and apoptosis, enhances activation of autophagy and increased cellular death		Vacuolating cytotoxin	[39,40,41,42,45]	[27,29,32,38,41,65,66]
**Other**					
katA/HP0875	antioxidant enzyme, neutralization of H_2_O_2_ and NaClO			[36,38,39,40,41]	enzyme activity determined in [62]
hcpD/HP0160	beta-lactamase		Putative solenoid proteins	[39,40,41]	
hcpA/HP0211	beta-lactamase, cysteine-rich 28 kD protein			[39,40,42]	
hcpE/HP0235	beta-lactamase			[39,40,41,42]	
hcpC/HP1098	beta-lactamase, cysteine-rich protein C			[38,39,40,42]	
tolB/HP1126	periplasmic protein interacting with outer membrane proteins (OMPs)			[38,39,40,41,42]	
csd3/HP0506	conserved hypothetical secreted protein		Secreted proteases	[39,41]	
ymxG/HP0657	processing protease			[38,39,41,42]	
pqqE/HP1012	metalloendopeptidase			[38,39,40,41,42,45]	
htrA/HP1018-9	acts as protease, degrades misfolded proteins and tight junction protein enabling delivery of CagA			[38,39,40,41,42]	
HP1037	metal ion binding aminopeptidase			[39,41]	
ggt/HP1118	transpeptidation and amino acid synthesis, enhances cell apoptosis, inhibits cellular proliferation			[38,39,40,41,42]	
ctpB/HP1350	serine-type endopeptidase			[38,39,40,41]	

Several uncharacterized proteases (HP0506, HP0657, HP1012, HP1037, HP1350) secreted by *H. pylori* were identified in OMVs (Table 2). Further, the activity of unidentified gelatinolytic proteases (76 kD and 96 kD) was observed in OMVs isolated from *H. pylori* 60190 cultured under iron-limiting conditions [72]. Among the proteases, the chaperone and serine protease high temperature requirement A (HtrA) has also been detected. HtrA was identified by mass spectroscopy and Western blot analysis in *H. pylori* CCUG 17874 OMVs, and its relative amount was higher than in the OM fraction [41]. HtrA was also identified in OMVs from *H. pylori* J99 and NCTC 11637 [38], *H. pylori* 251 and its *Δtol* or *Δpal* deletion mutants [40,42]. In recent years, HtrA has been intensively investigated as a secreted *H. pylori* factor that disrupts adherens junctions by targeting the cell adhesion protein and tumor suppressor E-cadherin of gastric epithelial cells [75]. Recently, occludin and claudin-8 were identified as additional substrates for HtrA, suggesting that tight junction complexes can be opened by HtrA as well [76]. Functionally, HtrA enables *H. pylori* to transmigrate across the epithelial layer, allowing the direct interaction with integrin-β1 at basolateral membranes so as to inject CagA into the polarized gastric epithelial cells [75,76]. While the HtrA substrate E-cadherin is cleaved within its extracellular domain, it was an interesting finding that the occludin and claudin-8 cleavage sites were identified in their intracellular domains [76]. Therefore, the concept of OMV-delivered and released virulence factors of *H. pylori* would explain how HtrA can target occludin and claudin-8 intracellularly. 

Another important factor in *H. pylori*-mediated pathogenesis is γ-glutamyltransferase (GGT), which has been identified in OMVs originating from *H. pylori* CCUG 17874 [41], *H. pylori* J99 and NCTC 11637 [38], and *H. pylori* 251 [40,42]. The presence of GGT was also confirmed in OMVs isolated from *H. suis* [77]. GGT is a secreted enzyme that converts glutamine into glutamate and ammonia, and converts glutathione into glutamate and cysteinyl glycine. *H. pylori* GGT and its catalyzed products have been implicated in cell cycle arrest and apoptosis. Further, *H. pylori* GGT can mediate immune tolerance by inhibiting T cell proliferation and modulating dendritic cell activation to give rise to regulatory T cell responses [24]. It is intriguing to speculate that OMVs contribute to the transport of the immunomodulatory *H. pylori* factors (e.g., VacA and GGT) across the epithelium and directly interact with immune cells.

**Figure 3:**
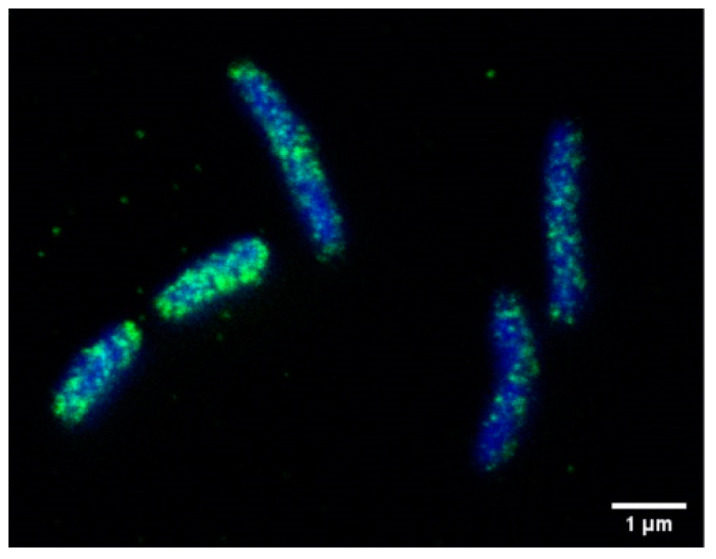
Localization of CagA in *H. pylori*. CagA (green) was detected using an anti-CagA antibody and analyzed by STED microscopy. Bacterial DNA was stained using DAPI (blue). Bar, 1 µM.

One of the best characterized virulence factors is the bacterial effector and oncoprotein CagA, which is translocated via a specialized type-IV secretion system (T4SS) into the cytoplasm of infected host cells [13,24,78]. Translocated CagA has a molecular weight of 135 kD, and is rapidly tyrosine phosphorylated in glutamate-proline-isoleucine-tyrosine-alanine (EPIYA) motifs in its carboxy-terminal part by host cell kinases of the c-Src and c-Abl families [79,80,81,82,83]. Both phospho- and non-phospho-CagA interact with distinct signaling proteins, which alters signaling and cell functions [84]. The observed changes include inflammatory phenotypes, altered proliferation, de-differentiation and elevated migratory capacity. Although in individual reports CagA was not detected in the OMV fraction from *cag*PAI-positive strains [27], there is a number of publications reporting CagA in OMVs as identified by mass spectroscopy [38,39,41,42,45]. Analyzing CagA in *H. pylori* by STED (Stimulated Emission Depletion) microscopy revealed CagA localized in speckles resembling OMVs (Figure 3), underlining the previous reports. The CagA level seemed to be similar in *H. pylori* CCUG 17874 OMVs and OM fractions, and it is associated with the surface of *H. pylori* vesicles. The presence of CagA was also confirmed by Western blotting in OMVs of *H. pylori* HP99 [29], *H. pylori* CCUG 17874 [41] and *H. pylori* NCTC 11637 [38]. CagA was detectable in serum-derived exosomes, leading to the hypothesis that those exosomes serve as nanocarriers for virulence factors and could be a causative mechanism through which *H. pylori* infection promotes extragastric diseases [85] (Figure 2). A possible function of OMV-delivered CagA might be indicated in a recent study using OMVs from *cagA*-positive and *cagA*-negative *H. pylori*. OMVs from *cagA*-positive bacteria induced an enhanced HO-1 expression in dendritic cells via the activation of nuclear factor kappa B (NF-kB) and nuclear factor erythroid 2-related factor 2 (Nrf2) [86]. *H. pylori* OMVs containing CagA were found to localize to the cell junctions, and were implicated in zonula occludens 1 (ZO-1) redistribution [87]. While the T4SS-translocated CagA drastically changes the motogenic response and cellular morphology of infected gastric epithelial cells, the incubation of AGS cells with these vesicles did not induce changes in morphology [56], which is in contrast to serum-derived OMVs [85]. Since CagA-mediated cell elongation is strictly dependent on CagA tyrosine phosphorylation [82], detailed analyses of OMV-delivered CagA and the activity of CagA kinases are necessary. 

### 4.3. Do H. pylori OMVs Modulate Host Immune Regulation?

Although the content of *H. pylori*-derived OMVs has been extensively analyzed, the functional consequences of OMVs’ interactions with host cells or *H. pylori* bacterial cells are largely unknown. Importantly, the gastric epithelial layer also recruits and activates cells’ immune system by the production and release of pro-inflammatory cytokines. As such, it is an interesting finding that *H. pylori*-derived OMVs induce IL-8 secretion by gastric epithelial AGS cells [40]. Corresponding to this observation, *H. pylori* NCTC 11637 OMVs induced the secretion of IL-8 from AGS cells, colonic epithelial T84 cells and duodenal explants [38]. OMVs induced IL-8 in a dose-dependent manner, which was shown to be independent of LPS [29]. Furthermore, IL-8 secretion was more pronounced using OMVs from *cag*PAI-negative Tx-30a compared to *cag*PAI-positive *H. pylori* strain 60190 [22]. This is in line with infection experiments investigating IL-8 secretion, which is clearly dependent on a functional T4SS, but independent of CagA translocation [88]. The sncRNAs identified in *H. pylori* strain J99 OMVs reduced IL-8 cytokine secretion [53], whereas it will be an interesting question to assess whether this might be mediated through a direct targeting of the IL-8 mRNA by the sncRNAs, and potential induction of RNA silencing. The fact that IL-8 is released in response to OMVs is interesting since it was further shown that OMVs also contain peptidoglycan (PG). Previously, PG has been identified to be translocated into *H. pylori*-infected cells via the T4SS, where it binds to the nucleotide-binding oligomerization domain-containing 1 (Nod1) protein and transactivates NF-κB-dependent pro-inflammatory genes [89]. Correspondingly, OMV derived PG has been described as an important factor in NF-κB-mediated IL-8 and CXCL2 (chemokine C-X-C motif ligand 2, macrophage inflammatory protein 2-alpha (MIP2-alpha)) induction in human and murine gastric cells, independent of the presence of proteins [20]. These data support the hypothesis that PG can induce NF-kB via OMVs independent of the T4SS. Recently, the *H. pylori* metabolite ADP heptose has been characterized as the key T4SS effector for the regulation of innate immune pathways [90,91]. Whether ADP heptose is also present in OMVs has not been analyzed yet. However, *H. pylori* OMVs can also induce NOD1 signaling, as shown by receptor-interacting protein (RIP2)-dependent autophagy prior to the release of IL-8 [58]. In summary, these data suggest that OMVs can induce the expression and release of proinflammatory cytokines and chemokines attracting immune cells to the place of infection.

Since molecules involved in immune evasion are also present in *H. pylori* OMVs, it was speculated that OMVs directly interfere with immune cell functions. As the fucosyltransferases (FutA and FutB) involved in lipopolysaccharide Lewis antigens modification were identified, as well were immune modulators neutrophil-activating protein (NapA) and outer inflammatory protein (OipA) (Table 2) [38,39,40,41,42], it would be interesting to investigate the biodistribution of OMVs and possible functional consequences of these factors for the immune response. A general effect of *H. pylori*-derived OMVs was also investigated on eosinophils isolated from the peripheral blood of human volunteers. *H. pylori* 60190 OMVs containing VacA led to cytoplasmic degranulation, increased CD11 and intracellular adhesion molecule (ICAM-1) expression, and to the release of the eosinophil cationic protein (ECP), which is a cytotoxic protein contributing to eosinophilic inflammation [92]. These OMVs were also used to study the response of peripheral blood mononuclear cells (PBMCs), whereby they strongly stimulated the release of IL-10 and IL-6, as well as a proliferative response, independent of VacA. OMVs were also tested on the Jurkat T cell line, where it caused pro- and anti-inflammatory responses, but also a loss of metabolic activity and apoptosis, which was promoted by VacA [65]. This observation is in contrast with a recent report describing that OMV treatment resulted in significant inhibition of Jurkat T cell proliferation, but did not induce apoptosis. In monocytes, *H. pylori* 60190 OMVs induced IL-10 and COX-2 expression [93]. OMVs isolated from *H. pylori* HP99 culture were shown to induce in vivo production of IL-6 in mouse macrophages, which was LPS-independent [29]. *H. pylori* 60190 OMVs induced expression of heme oxygenase-1 (HO-1) in bone marrow-derived mouse dendritic cells. HO-1 is also implicated in inflammation and oxidative damage. A such the authors speculated that upregulated HO-1 expression protects cells from immunopathogenesis or stress damage [86].

The effect of OMVs on the adaptive immune response was also investigated in vivo. The intragastric administration of *H. pylori* 60190 OMVs led to a production of specific IgG in mice [94,95]. Intragastric immunization was NOD1-dependent but independent of TLR signaling [20]. Furthermore, cytokine levels like IFN-γ, IL-17 and IL-4 were elevated in mouse spleens [29]. Immunization with *H. pylori* 60190 OMVs induced antibody reactivity against an 18 kD protein [26,94], which was later recognized as a specific anti-lipoprotein 20 (Lpp20, HP1456) response. Interestingly, animals transplanted with hybridoma tumors producing antibodies against *H. pylori* Lpp20 showed lower numbers of *H. pylori* colonizing the stomach, suggesting Lpp20 as a vaccine candidate [95]. In fact, mouse immunization studies with OMVs or recombinant Lpp20 revealed significantly lower levels of colonization in immunized animals [96].

## 5. Conclusions

Knowledge about the fate and function of OMVs in *H. pylori* pathogenesis is still at its beginning, but accumulating data point to its functional role in bacterial pathogenesis through attachment to, and possible uptake into, host cells (Figure 2). In this context, reliable and unbiased methods are required to analyze OMV internalization and the fate of OMVs and their cargo inside the host cells. It is an attractive hypothesis that OMVs are transported through the epithelial monolayer and are released at the basal membrane, which could explain the delivery of virulence factors to extragastric body sites (Figure 2). Additionally, the idea that the OMV content is released into the host cell´s cytosol is a fascinating novel concept concerning how *H. pylori* might interfere with host cell function, and needs to be investigated in future. 

On the basis of their emerging pathophysiological functions in transporting functional macromolecules across biological barriers and kingdoms, both into other enteric bacteria as well as into cells and tissues of the mammalian host, it is intriguing to speculate about the potential biomedical applications of *H. pylori* OMVs. Beyond targeting the epithelium, the biodistribution of *H. pylori* OMVs in the human body has not been systematically documented to date. Due to the induction of mucosal barrier instability and their small size, it is conceivable that OMVs cannot only penetrate deeper layers of the gastric mucosa but potentially also enter the circulation. It was shown that CagA is present in vesicles derived from the serum of patients infected with *cagA* positive strains [85], suggesting that either OMVs themselves or secondary exosomes secreted from mucosal cells targeted by OMVs might enter the blood stream. If this holds true, this would open up a new avenue for vesicle-based diagnostics and biomarkers of *H. pylori* infection. Secondly, like extracellular vesicles from mammalian sources, OMVs are also of potential interest as a biological drug delivery vehicle due to their effectiveness in penetrating biological barriers, entering their recipient cells in a targeted manner to functionally deliver biological macromolecules to distant sites while evading (or modulating) the immune system (reviewed in [97]) (Figure 2). The ease of bioengineering the vesicles by genetic modification of the bacterial cells, as well as the relative ease of production at scale, makes OMVs a potentially attractive drug delivery platform. Their immunogenicity, as well as their similarity to their bacterial mother cells, might however make them of particular interest for vaccine development [61]. In fact, a *Neisseria meningitidis* OMV-containing vaccine (Bexsero) was approved in 2013 for meningitis B by the FDA and EMA, and further OMV based vaccines are currently in development for other pathogenic bacteria [17,98,99,100]. Vaccine development for *H. pylori* infection has historically been challenging. A recently published immunization study with oral administration of *H. pylori* OMVs in mice, however, has shown strong protective effects against *H. pylori* infection via a Th2 driven immune response [45], indicating that OMV-based *H. pylori* vaccines for clinical testing could eventually appear on the horizon. Finally, it has been proposed that OMVs of pathogenic bacteria might be used as biological antibiotics against other infectious pathogens; OMVs from *Borrelia* [101], *E. coli* [102,103], *Actinomyces* [104] or *Helicobacter* [51] have been used to deliver cytotoxic proteins or peptides, resulting in lysis and cell death, and it has been proposed that this activity is targeted to other bacteria rather than the host. This fascinating strategy of using one pathogenic microorganism to not only cross-regulate its host but also produce its own ‘predatory microvesicle artillery’ [97] to defend itself against other pathogenic species competing for the same niche might open up a fundamentally new principle for next generation antibiotics. 

## Figures and Tables

**Table 1:** Size of *H. pylori* OMVs.

Strain	Size in Diameter	Reference
26695 (ATCC 700392)	50–500 nm	[39]
CCUG 17874 (ATCC 11637)	20–300 nm	[19,31,32,41]
60190 (ATCC 49503)	50–300 nm	[19]
J99 (ATCC 700824)	20–200 nm	[38]
7.13	100–200 nm	[45]
251	20–400 nm	[40,42]
H99	25–200 nm	[29]

## References

[1] Piazuelo M.B., Epplein M., Correa P. (2010). Gastric Cancer: An Infectious Disease. Infect. Dis. Clin. N. Am..

[2] Waskito L.A., Salama N.R., Yamaoka Y. (2018). Pathogenesis of Helicobacter pylori infection. Helicobacter.

[3] Venerito M., Vasapolli R., Rokkas T., Malfertheiner P. (2018). Gastric cancer: Epidemiology, prevention, and therapy. Helicobacter.

[4] (1994). Schistosomes, liver flukes and Helicobacter pylori. IARC Working Group on the Evaluation of Carcinogenic Risks to Humans. Lyon, 7–14 June 1994. IARC Monogr. Eval. Carcinog. Risks Hum..

[5] Parkin D.M. (2006). The global health burden of infection-associated cancers in the year 2002. Int. J. Cancer.

[6] Moss S.F. (2017). The Clinical Evidence Linking Helicobacter pylori to Gastric Cancer. Cell. Mol. Gastroenterol. Hepatol..

[7] Correa P., Piazuelo M.B. (2011). The gastric precancerous cascade. J. Dig. Dis..

[8] Correa P. (1992). Human gastric carcinogenesis: A multistep and multifactorial process—First American Cancer Society Award Lecture on Cancer Epidemiology and Prevention. Cancer Res..

[9] Choi I.J., Kook M.-C., Kim Y.-I., Cho S.-J., Lee J.Y., Kim C.G., Park B., Nam B.H. (2018). Helicobacter pylori Therapy for the Prevention of Metachronous Gastric Cancer. N. Engl. J. Med..

[10] Ford A.C., Forman D., Hunt R.H., Yuan Y., Moayyedi P. (2014). Helicobacter pylori eradication therapy to prevent gastric cancer in healthy asymptomatic infected individuals: Systematic review and meta-analysis of randomised controlled trials. BMJ.

[11] Šterbenc A., Jarc E., Poljak M., Homan M. (2019). Helicobacter pylori virulence genes. World J. Gastroenterol..

[12] Posselt G., Backert S., Wessler S. (2013). The functional interplay of Helicobacter pylori factors with gastric epithelial cells induces a multi-step process in pathogenesis. Cell Commun. Signal..

[13] Backert S., Clyne M. (2011). Pathogenesis of Helicobacter pylori Infection. Helicobacter.

[14] Zhang X.-Y., Zhang P.-Y., Aboul-Soud M.A. (2016). From inflammation to gastric cancer: Role of Helicobacter pylori. Oncol. Lett..

[15] Deatherage B.L., Cookson B.T. (2012). Membrane Vesicle Release in Bacteria, Eukaryotes, and Archaea: A Conserved yet Underappreciated Aspect of Microbial Life. Infect. Immun..

[16] Malloci M., Perdomo L., Veerasamy M., Andriantsitohaina R., Simard G., Martínez M.C. (2019). Extracellular Vesicles: Mechanisms in Human Health and Disease. Antioxid. Redox Signal..

[17] Bitto N.J., Kaparakis-Liaskos M. (2017). The Therapeutic Benefit of Bacterial Membrane Vesicles. Int. J. Mol. Sci..

[18] Schwechheimer C., Kuehn M.J. (2015). Outer-membrane vesicles from Gram-negative bacteria: Biogenesis and functions. Nat. Rev. Genet..

[19] Fiocca R., Necchi V., Sommi P., Ricci V., Telford J., Cover T.L., Solcia E. (1999). Release of Helicobacter pylori vacuolating cytotoxin by both a specific secretion pathway and budding of outer membrane vesicles. Uptake of released toxin and vesicles by gastric epithelium. J. Pathol..

[20] Kaparakis M., Turnbull L., Carneiro L., Firth S., Coleman H.A., Parkington H.C., Le Bourhis L., Karrar A., Viala J., Mak J. (2010). Bacterial membrane vesicles deliver peptidoglycan to NOD1 in epithelial cells. Cell. Microbiol..

[21] Shen Y., Torchia M.L.G., Lawson G.W., Karp C.L., Ashwell J.D., Mazmanian S.K. (2012). Outer membrane vesicles of a human commensal mediate immune regulation and disease protection. Cell Host Microbe.

[22] Ismail S., Hampton M.B., Keenan J. (2003). Helicobacter pylori Outer Membrane Vesicles Modulate Proliferation and Interleukin-8 Production by Gastric Epithelial Cells. Infect. Immun..

[23] Parker H., Keenan J.I. (2012). Composition and function of Helicobacter pylori outer membrane vesicles. Microbes Infect..

[24] Sgouras D., Tegtmeyer N., Wessler S. (2019). Activity and Functional Importance of Helicobacter pylori Virulence Factors. Adv. Exp. Med. Biol..

[25] Wessler S., Backert S. (2008). Molecular mechanisms of epithelial-barrier disruption by Helicobacter pylori. Trends Microbiol..

[26] Keenan J.I., Allardyce R.A., Bagshaw P.F. (1997). Dual silver staining to characterise Helicobacter spp. outer membrane components. J. Immunol. Methods.

[27] Keenan J., Day T., Neal S., Cook B., Perez-Perez G., Allardyce R., Bagshaw P. (2000). A role for the bacterial outer membrane in the pathogenesis of Helicobacter pylori infection. FEMS Microbiol. Lett..

[28] Hynes S.O., Keenan J.I., Ferris J.A., Annuk H., Moran A.P. (2005). Lewis Epitopes on Outer Membrane Vesicles of Relevance to Helicobacter pylori Pathogenesis. Helicobacter.

[29] Choi H.-I., Choi J.-P., Seo J., Kim B.J., Rho M., Han J.K., Kim J.G. (2017). Helicobacter pylori-derived extracellular vesicles increased in the gastric juices of gastric adenocarcinoma patients and induced inflammation mainly via specific targeting of gastric epithelial cells. Exp. Mol. Med..

[30] Grande R., Di Marcantonio M.C., Robuffo I., Pompilio A., Celia C., Di Marzio L., Paolino D., Codagnone M., Muraro R., Stoodley P. (2015). Helicobacter pylori ATCC 43629/NCTC 11639 Outer Membrane Vesicles (OMVs) from Biofilm and Planktonic Phase Associated with Extracellular DNA (eDNA). Front. Microbiol..

[31] Heczko U., Smith V.C., Meloche R.M., Buchan A.M.J., Finlay B.B. (2000). Characteristics of Helicobacter pylori attachment to human primary antral epithelial cells. Microbes Infect..

[32] Sommi P., Ricci V., Fiocca R., Necchi V., Romano M., Telford J.L., Solcia E., Ventura U. (1998). Persistence of Helicobacter pylori VacA toxin and vacuolating potential in cultured gastric epithelial cells. Am. J. Physiol. Content.

[33] Yonezawa H., Osaki T., Woo T., Kurata S., Zaman C., Hojo F., Hanawa T., Kato S., Kamiya S. (2011). Analysis of outer membrane vesicle protein involved in biofilm formation of Helicobacter pylori. Anaerobe.

[34] Yonezawa H., Osaki T., Fukutomi T., Hanawa T., Kurata S., Zaman C., Hojo F., Kamiya S. (2016). Diversification of the AlpB Outer Membrane Protein of Helicobacter pylori Affects Biofilm Formation and Cellular Adhesion. J. Bacteriol..

[35] Yonezawa H., Osaki T., Kurata S., Fukuda M., Kawakami H., Ochiai K., Hanawa T., Kamiya S. (2009). Outer Membrane Vesicles of Helicobacter pylori TK1402 are Involved in Biofilm Formation. BMC Microbiol..

[36] Ronci M., Del Prete S., Puca V., Carradori S., Carginale V., Muraro R., Mincione G., Aceto A., Sisto F., Supuran C.T. (2019). Identification and characterization of the α-CA in the outer membrane vesicles produced by Helicobacter pylori. J. Enzym. Inhib. Med. Chem..

[37] Rozo A.J., Cox M.H., Devitt A., Rothnie A.J., Goddard A.D. (2020). Biophysical analysis of lipidic nanoparticles. Methods.

[38] Mullaney E., Brown P., Smith S.M., Botting C.H., Yamaoka Y., Terres A.M., Kelleher D.P., Windle H.J. (2009). Proteomic and functional characterization of the outer membrane vesicles from the gastric pathogen Helicobacter pylori. Proteom. Clin. Appl..

[39] Zavan L., Bitto N.J., Johnston E.L., Greening D.W., Kaparakis-Liaskos M. (2018). Helicobacter pylori Growth Stage Determines the Size, Protein Composition, and Preferential Cargo Packaging of Outer Membrane Vesicles. Proteomics.

[40] Turner L., Bitto N.J., Steer D.L., Lo C., D’Costa K., Ramm G., Shambrook M., Hill A.F., Ferrero R.L., Kaparakis-Liaskos M. (2018). Helicobacter pylori Outer Membrane Vesicle Size Determines Their Mechanisms of Host Cell Entry and Protein Content. Front. Immunol..

[41] Olofsson A., Vallström A., Petzold K., Tegtmeyer N., Schleucher J., Carlsson S., Haas R., Backert S., Wai S.N., Gröbner G. (2010). Biochemical and functional characterization of Helicobacter pylori vesicles. Mol. Microbiol..

[42] Turner L., Praszkier J., Hutton M.L., Steer D., Ramm G., Kaparakis-Liaskos M., Ferrero R.L. (2015). Increased Outer Membrane Vesicle Formation in a Helicobacter pylori tolB Mutant. Helicobacter.

[43] Penfold C.N., Li C., Zhang Y., Vankemmelbeke M., James R. (2012). Colicin A binds to a novel binding site of TolA in the Escherichia coli periplasm. Biochem. Soc. Trans..

[44] Godlewska R., Wiśniewska K., Pietras Z., Jagusztyn-Krynicka E.K. (2009). Peptidoglycan-associated lipoprotein (Pal) of Gram-negative bacteria: Function, structure, role in pathogenesis and potential application in immunoprophylaxis. FEMS Microbiol. Lett..

[45] Liu Q., Li X., Zhang Y., Song Z., Li R., Ruan H., Huang X. (2019). Orally-administered outer-membrane vesicles from Helicobacter pylori reduce H. pylori infection via Th2-biased immune responses in mice. Pathog. Dis..

[46] Yonezawa H., Osaki T., Kamiya S. (2015). Biofilm Formation by Helicobacter pylori and Its Involvement for Antibiotic Resistance. BioMed. Res. Int..

[47] Carron M.A., Tran V.R., Sugawa C., Coticchia J.M. (2006). Identification of Helicobacter pylori Biofilms in Human Gastric Mucosa. J. Gastrointest. Surg..

[48] Coticchia J., Sugawa C., Tran V., Gurrola J., Kowalski E., Carron M. (2006). Presence and Density of Helicobacter pylori Biofilms in Human Gastric Mucosa in Patients with Peptic Ulcer Disease. J. Gastrointest. Surg..

[49] Skotland T., Sandvig K., Llorente A. (2017). Lipids in exosomes: Current knowledge and the way forward. Prog. Lipid Res..

[50] Keenan J.I., Davis K.A., Beaugie C.R., McGovern J.J., Moran A.P. (2008). Alterations in Helicobacter pylori outer membrane and outer membrane vesicle-associated lipopolysaccharides under iron-limiting growth conditions. Innate Immun..

[51] Koeppen K., Hampton T.H., Jarek M., Scharfe M., Gerber S.A., Mielcarz D.W., Demers E.G., Dolben E.L., Hammond J.H., Hogan D.A. (2016). A Novel Mechanism of Host-Pathogen Interaction through sRNA in Bacterial Outer Membrane Vesicles. PLoS Pathog..

[52] Polakovicova I., Jerez S., Wichmann I.A., Sandoval-Bórquez A., Carrasco-Véliz N., Corvalán A.L. (2018). Role of microRNAs and Exosomes in Helicobacter pylori and Epstein-Barr Virus Associated Gastric Cancers. Front. Microbiol..

[53] Zhang H., Zhang Y., Song Z., Li R., Ruan H., Liu Q., Huang X. (2020). sncRNAs packaged by Helicobacter pylori outer membrane vesicles attenuate IL-8 secretion in human cells. Int. J. Med. Microbiol..

[54] Lee H.-J. (2019). Microbe-Host Communication by Small RNAs in Extracellular Vesicles: Vehicles for Transkingdom RNA Transportation. Int. J. Mol. Sci..

[55] Patton J.G., Franklin J.L., Weaver A.M., Vickers K., Zhang B., Coffey R.J., Ansel K.M., Blelloch R., Goga A., Huang B. (2015). Biogenesis, delivery, and function of extracellular RNA. J. Extracell. Vesicles.

[56] Parker H., Chitcholtan K., Hampton M.B., Keenan J.I. (2010). Uptake of Helicobacter pylori Outer Membrane Vesicles by Gastric Epithelial Cells. Infect. Immun..

[57] Olofsson A., Skalman L.N., Obi I., Lundmark R., Arnqvist A. (2014). Uptake of Helicobacter pylori Vesicles Is Facilitated by Clathrin-Dependent and Clathrin-Independent Endocytic Pathways. mBio.

[58] Irving A.T., Mimuro H., Kufer T.A., Lo C.Y., Wheeler R., Turner L., Thomas B.J., Malosse C., Gantier M.P., Casillas L.N. (2014). The Immune Receptor NOD1 and Kinase RIP2 Interact with Bacterial Peptidoglycan on Early Endosomes to Promote Autophagy and Inflammatory Signaling. Cell Host Microbe.

[59] Heusermann W., Hean J., Trojer D., Steib E., Von Bueren S., Graff-Meyer A., Genoud C., Martin K., Pizzato N., Voshol J. (2016). Exosomes surf on filopodia to enter cells at endocytic hot spots, traffic within endosomes, and are targeted to the ER. J. Cell Biol..

[60] Chitcholtan K., Hampton M.B., Keenan J.I. (2008). Outer membrane vesicles enhance the carcinogenic potential of Helicobacter pylori. Carcinogenesis.

[61] Van Der Pol L., Stork M., Van Der Ley P. (2015). Outer membrane vesicles as platform vaccine technology. Biotechnol. J..

[62] Lekmeechai S., Su Y.-C., Brant M., Alvarado-Kristensson M., Vallström A., Obi I., Arnqvist A., Riesbeck K. (2018). Helicobacter pylori Outer Membrane Vesicles Protect the Pathogen From Reactive Oxygen Species of the Respiratory Burst. Front. Microbiol..

[63] Eaton K.A., Brooks C.L., Morgan D.R., Krakowka S. (1991). Essential role of urease in pathogenesis of gastritis induced by Helicobacter pylori in gnotobiotic piglets. Infect. Immun..

[64] Hathroubi S., Servetas S.L., Windham I., Merrell D.S., Ottemann K.M. (2018). Helicobacter pylori Biofilm Formation and Its Potential Role in Pathogenesis. Microbiol. Mol. Biol. Rev..

[65] Winter J., Letley D., Rhead J., Atherton J., Robinson K. (2014). Helicobacter pylori Membrane Vesicles Stimulate Innate Pro- and Anti-Inflammatory Responses and Induce Apoptosis in Jurkat T Cells. Infect. Immun..

[66] Ayala G., Torres L., Espinosa M., Fierros-Zarate G., Maldonado V., Melendez-Zajgla J. (2006). External membrane vesicles from Helicobacter pylori induce apoptosis in gastric epithelial cells. FEMS Microbiol. Lett..

[67] Ilver D., Barone S., Mercati D., Lupetti P., Telford J.L. (2004). Helicobacter pylori toxin VacA is transferred to host cells via a novel contact-dependent mechanism. Cell. Microbiol..

[68] Molinari M., Galli C., Norais N., Telford J.L., Rappuoli R., Luzio J.P., Montecucco C. (1997). Vacuoles Induced by Helicobacter pylori Toxin Contain Both Late Endosomal and Lysosomal Markers. J. Biol. Chem..

[69] Wang Y.-H., Wu J.-J., Lei H.-Y. (2009). The Autophagic Induction in Helicobacter pylori-Infected Macrophage. Exp. Biol. Med..

[70] Galmiche A., Rassow J., Doye A., Cagnol S., Chambard J.C., Contamin S., De Thillot V., Just I., Ricci V., Solcia E. (2000). The N-terminal 34 kDa fragment of Helicobacter pylori vacuolating cytotoxin targets mitochondria and induces cytochrome c release. EMBO J..

[71] Papini E., Satin B., Norais N., De Bernard M., Telford J.L., Rappuoli R., Montecucco C. (1998). Selective increase of the permeability of polarized epithelial cell monolayers by Helicobacter pylori vacuolating toxin. J. Clin. Investig..

[72] Keenan J., Allardyce R.A. (2000). Iron influences the expression of Helicobacter pylori outer membrane vesicle-associated virulence factors. Eur. J. Gastroenterol. Hepatol..

[73] Ricci V., Chiozzi V., Necchi V., Oldani A., Romano M., Solcia E., Ventura U. (2005). Free-soluble and outer membrane vesicle-associated VacA from Helicobacter pylori: Two forms of release, a different activity. Biochem. Biophys. Res. Commun..

[74] Yamasaki E., Wada A., Kumatori A., Nakagawa I., Funao J., Nakayama M., Hisatsune J., Kimura M., Moss J., Hirayama T. (2006). Helicobacter pylori Vacuolating Cytotoxin Induces Activation of the Proapoptotic Proteins Bax and Bak, Leading to Cytochrome c Release and Cell Death, Independent of Vacuolation. J. Biol. Chem..

[75] Hoy B., Löwer M., Weydig C., Carra G., Tegtmeyer N., Geppert T., Schröder P., Sewald N., Backert S., Schneider G. (2010). Helicobacter pylori HtrA is a new secreted virulence factor that cleaves E-cadherin to disrupt intercellular adhesion. EMBO Rep..

[76] Tegtmeyer N., Wessler S., Necchi V., Rohde M., Harrer A., Rau T.T., Asche C.I., Boehm M., Loessner H., Figueiredo C. (2017). Helicobacter pylori Employs a Unique Basolateral Type IV Secretion Mechanism for CagA Delivery. Cell Host Microbe.

[77] Zhang G., Ducatelle R., Pasmans F., D’Herde K., Huang L., Smet A., Haesebrouck F., Flahou B. (2013). Effects of Helicobacter suis γ-Glutamyl Transpeptidase on Lymphocytes: Modulation by Glutamine and Glutathione Supplementation and Outer Membrane Vesicles as a Putative Delivery Route of the Enzyme. PLoS ONE.

[78] Hatakeyama M. (2004). Oncogenic mechanisms of the Helicobacter pylori CagA protein. Nat. Rev. Cancer.

[79] Poppe M., Feller S.M., Romer G., Wessler S. (2006). Phosphorylation of Helicobacter pylori CagA by c-Abl leads to cell motility. Oncogene.

[80] Mueller D., Tegtmeyer N., Brandt S., Yamaoka Y., De Poire E., Sgouras D., Wessler S., Torres J., Smolka A., Backert S. (2012). c-Src and c-Abl kinases control hierarchic phosphorylation and function of the CagA effector protein in Western and East Asian Helicobacter pylori strains. J. Clin. Investig..

[81] Krisch L.M., Posselt G., Hammerl P., Wessler S. (2016). CagA Phosphorylation in Helicobacter pylori-Infected B Cells Is Mediated by the Nonreceptor Tyrosine Kinases of the Src and Abl Families. Infect. Immun..

[82] Selbach M., Moese S., Hauck C.R., Meyer T.F., Backert S. (2002). Src Is the Kinase of the Helicobacter pylori CagA Protein in Vitro and in Vivo. J. Biol. Chem..

[83] Stein M., Bagnoli F., Halenbeck R., Rappuoli R., Fantl W.J., Covacci A. (2002). c-Src/Lyn kinases activate Helicobacter pylori CagA through tyrosine phosphorylation of the EPIYA motifs. Mol. Microbiol..

[84] Selbach M., Paul F.E., Brandt S., Guye P., Daumke O., Backert S., Dehio C., Mann M. (2009). Host Cell Interactome of Tyrosine-Phosphorylated Bacterial Proteins. Cell Host Microbe.

[85] Shimoda A., Ueda K., Nishiumi S., Murata-Kamiya N., Mukai S.-A., Sawada S.-I., Azuma T., Hatakeyama M., Akiyoshi K. (2016). Exosomes as nanocarriers for systemic delivery of the Helicobacter pylori virulence factor CagA. Sci. Rep..

[86] Ko S.H., Rho D.J., Jeon J.I., Kim Y.-J., Woo H.A., Kim N., Kim J.M. (2016). Crude Preparations of Helicobacter pylori Outer Membrane Vesicles Induce Upregulation of Heme Oxygenase-1 via Activating Akt-Nrf2 and mTOR–IκB Kinase–NF-κB Pathways in Dendritic Cells. Infect. Immun..

[87] Turkina M.V., Olofsson A., Magnusson K.-E., Arnqvist A., Vikström E. (2015). Helicobacter pylori vesicles carrying CagA localize in the vicinity of cell–cell contacts and induce histone H1 binding to ATP in epithelial cells. FEMS Microbiol. Lett..

[88] Fischer W., Püls J., Buhrdorf R., Gebert B., Odenbreit S., Haas R. (2002). Systematic mutagenesis of the Helicobacter pylori cag pathogenicity island: Essential genes for CagA translocation in host cells and induction of interleukin-8. Mol. Microbiol..

[89] Viala J., Chaput C., Boneca I.G., Cardona A., Girardin S.E., Moran A.P., Athman R., Mémet S., Huerre M.R., Coyle A.J. (2004). Nod1 responds to peptidoglycan delivered by the Helicobacter pylori cag pathogenicity island. Nat. Immunol..

[90] Pfannkuch L., Hurwitz R., Traulsen J., Sigulla J., Poeschke M., Matzner L., Kosma P., Schmid M., Meyer T.F. (2019). ADP heptose, a novel pathogen-associated molecular pattern identified in Helicobacter pylori. FASEB J..

[91] Zimmermann S., Pfannkuch L., Al-Zeer M.A., Bartfeld S., Koch M., Liu J., Rechner C., Soerensen M., Sokolova O., Zamyatina A. (2017). ALPK1- and TIFA-Dependent Innate Immune Response Triggered by the Helicobacter pylori Type IV Secretion System. Cell Rep..

[92] Ko S.H., Jeon J.I., Kim Y.-J., Yoon H.J., Kim H., Kim N., Kim J.S., Kim J.M. (2015). Helicobacter pylori Outer Membrane Vesicle Proteins Induce Human Eosinophil Degranulation via a β2 Integrin CD11/CD18- and ICAM-1-Dependent Mechanism. Mediat. Inflamm..

[93] Hock B.D., McKenzie J.L., Keenan J.I. (2017). Helicobacter pylori outer membrane vesicles inhibit human T cell responses via induction of monocyte COX-2 expression. Pathog. Dis..

[94] Keenan J.I., Allardyce R., Bagshaw P.F. (1998). Lack of protection following immunisation with H. pylori outer membrane vesicles highlights antigenic differences between *H. felis* and *H. pylori*. FEMS Microbiol. Lett..

[95] Keenan J., Oliaro J., Domigan N., Potter H., Aitken G., Allardyce R., Roake J. (2000). Immune Response to an 18-Kilodalton Outer Membrane Antigen Identifies Lipoprotein 20 as a Helicobacter pylori Vaccine Candidate. Infect. Immun..

[96] Keenan J., Rijpkema S., Durrani Z., Roake J.A. (2003). Differences in immunogenicity and protection in mice and guinea pigs following intranasal immunization with Helicobacter pylori outer membrane antigens. FEMS Immunol. Med. Microbiol..

[97] Jain S., Pillai J. (2017). Bacterial membrane vesicles as novel nanosystems for drug delivery. Int. J. Nanomed..

[98] Pathirana R.D., Kaparakis-Liaskos M. (2016). Bacterial membrane vesicles: Biogenesis, immune regulation and pathogenesis. Cell. Microbiol..

[99] Tan K., Li R., Huang X., Liu Q. (2018). Outer Membrane Vesicles: Current Status and Future Direction of These Novel Vaccine Adjuvants. Front. Microbiol..

[100] Irene C., Fantappiè L., Caproni E., Zerbini F., Anesi A., Tomasi M., Zanella I., Stupia S., Prete S., Valensin S. (2019). Bacterial outer membrane vesicles engineered with lipidated antigens as a platform for Staphylococcus aureus vaccine. Proc. Natl. Acad. Sci. USA.

[101] Toledo A., Coleman J.L., Kuhlow C.J., Crowley J.T., Benach J.L. (2011). The Enolase of Borrelia burgdorferi Is a Plasminogen Receptor Released in Outer Membrane Vesicles. Infect. Immun..

[102] Gankema H., Wensink J., Guinée P.A.M., Jansen W.H., Witholt B. (1980). Some characteristics of the outer membrane material released by growing enterotoxigenic *Escherichia coli*. Infect. Immun..

[103] Horstman A.L., Kuehn M.J. (2000). Enterotoxigenic Escherichia coli Secretes Active Heat-labile Enterotoxin via Outer Membrane Vesicles. J. Biol. Chem..

[104] Kato S., Kowashi Y., DeMuth D.R. (2002). Outer membrane-like vesicles secreted by Actinobacillus actinomycetemcomitans are enriched in leukotoxin. Microb. Pathog..

